# *Acidithiobacillus caldus* Sulfur Oxidation Model Based on Transcriptome Analysis between the Wild Type and Sulfur Oxygenase Reductase Defective Mutant

**DOI:** 10.1371/journal.pone.0039470

**Published:** 2012-09-12

**Authors:** Linxu Chen, Yilin Ren, Jianqun Lin, Xiangmei Liu, Xin Pang, Jianqiang Lin

**Affiliations:** 1 State Key Lab of Microbial Technology, Shandong University, Jinan, China; 2 School of Life Science, Shandong Normal University, Jinan, China; US Naval Reseach Laboratory, United States of America

## Abstract

**Background:**

*Acidithiobacillus caldus* (*A. caldus*) is widely used in bio-leaching. It gains energy and electrons from oxidation of elemental sulfur and reduced inorganic sulfur compounds (RISCs) for carbon dioxide fixation and growth. Genomic analyses suggest that its sulfur oxidation system involves a truncated sulfur oxidation (Sox) system (omitting SoxCD), non-Sox sulfur oxidation system similar to the sulfur oxidation in *A. ferrooxidans*, and sulfur oxygenase reductase (SOR). The complexity of the sulfur oxidation system of *A. caldus* generates a big obstacle on the research of its sulfur oxidation mechanism. However, the development of genetic manipulation method for *A. caldus* in recent years provides powerful tools for constructing genetic mutants to study the sulfur oxidation system.

**Results:**

An *A. caldus* mutant lacking the sulfur oxygenase reductase gene (*sor*) was created and its growth abilities were measured in media using elemental sulfur (S^0^) and tetrathionate (K_2_S_4_O_6_) as the substrates, respectively. Then, comparative transcriptome analysis (microarrays and real-time quantitative PCR) of the wild type and the *Δsor* mutant in S^0^ and K_2_S_4_O_6_ media were employed to detect the differentially expressed genes involved in sulfur oxidation. SOR was concluded to oxidize the cytoplasmic elemental sulfur, but could not couple the sulfur oxidation with the electron transfer chain or substrate-level phosphorylation. Other elemental sulfur oxidation pathways including sulfur diooxygenase (SDO) and heterodisulfide reductase (HDR), the truncated Sox pathway, and the S_4_I pathway for hydrolysis of tetrathionate and oxidation of thiosulfate in *A. caldus* are proposed according to expression patterns of sulfur oxidation genes and growth abilities of the wild type and the mutant in different substrates media.

**Conclusion:**

An integrated sulfur oxidation model with various sulfur oxidation pathways of *A. caldus* is proposed and the features of this model are summarized.

## Introduction

*Acidithiobacillus caldus* (*A. caldus*), a gram-negative, acidophilic, obligately chemolithotrophic, moderately thermophilic bacterium [1], [2] and an important member of a consortium of microorganisms used for industrial bioleaching [3], plays key roles together with iron-oxidizing bacteria in bio-leaching processes [4], [5]. *A. caldus* has the capability of oxidizing elemental sulfur and a wide range of reduced inorganic sulfur compounds (RISCs), but could not oxidize ferrous iron. It uses energy and electrons derived from sulfur oxidation for carbon dioxide fixation and other anabolic processes [1], [2], [6], [7].

Oxidation of elemental sulfur and RISCs happens normally in some chemolithotrophic bacteria and archaea [8]–[10]. The sulfur oxidizing (Sox) enzyme system in lithoautotrophic *Paracoccus pantotrophus* (*P. pantotrophus*), responsible for the oxidation of sulfide, elemental sulfur, thiosulfate, and sulfite to sulfate, accompanied by electron transfer to cytochrome c, has been well studied [8]–[12]. It is located in the periplasm and constituted generally by four proteins: SoxYZ, SoxAX, SoxB and Sox(CD)_2_ [13]. Initially, SoxAX initiates the oxidation of thiosulfate (S_2_O_3_^2−^) producing SoxY-thiocysteine-S-sulfate (SoxYZ-S-S-SO_3_^−^) [14]; secondly, SoxB hydrolyzes sulfate (SO_4_^2−^) from the thiocysteine-S-sulfate residue (SoxYZ-S-S-SO_3_^−^) producing S-thiocysteine (SoxYZ-S-S^−^); thirdly, Sox(CD)_2_ may oxidize the outer sulfur atom of S-thiocysteine producing SoxYZ-cysteine-S-sulfate (SoxYZ-S-SO_3_^−^); finally, sulfate is hydrolyzed and removed by SoxB from SoxYZ-S-SO_3_^−^, and SoxYZ is regenerated. However, Sox(CD)_2_ is absent in the so-called truncated Sox system of many prototypical α-Proteobacteria [8], [10]. Another sulfur oxidation system based on the sulfur oxygenase reductase (SOR) has been elaborated in several acidophilic and thermophilic archaea [10], [15]. SOR is able to catalyze the disproportionation of elemental sulfur, producing sulfite, thiosulfate, and sulfide [16], [17]. The reaction has several characteristics: (1) it takes place in the cytoplasm; (2) it is dioxygen (O_2_)-dependent with no external cofactors or electron donors required [16]; (3) sulfur oxidation is not coupled with electron transfer or substrate-level phosphorylation in *Acidianus ambivalens* (*A. ambivalens*) [18].

The sulfur oxidation system in acidophilic *Acidithiobacillus* genus is related to three representative species widely used in bio-leaching, which are *A. ferrooxidans*, *A. thiooxidans* and *A. caldus*. Although, the sulfur metabolic mechanism in *A. ferrooxidans* has been studied for many years, it is still not completely understood. The elemental sulfur in nature consists of a stable octasulfane ring (S_8_), which forms orthorhombic crystals insoluble in water [19]. An elemental sulfur activation and oxidation model was proposed in *Acidithiobacillus* [20], [21]. The S_8_ is firstly activated to become thiol-bound sulfane sulfur atoms (R-S-SH) and then transported into the periplasm where it is oxidized by sulfur dioxygenase (SDO) [20], [21]. SDO enzyme activity was detected from the crude cell extracts of *A. ferrooxidans* and *A. thiooxidans*, but SDO enzyme protein has not been purified and the gene(s) encoding for SDO activity not yet been identified [20], [22]. Another elemental sulfur oxidation enzyme in *A. ferrooxidans* is the cytoplasmic heterodisulfide reductase complex (HdrABC), but it is a speculation from the genomic and transcriptomic analysis, not from biochemical experimental data [23]. The S_4_I pathway of thiosulfate oxidation via the form of tetrathionate intermediates exists widely in *Acidithiobacillus*: the periplasmic thiosulfate is oxidized to tetrathionate by thiosulfate quinine oxidoreduetase (TQO); then, tetrathionate is hydrolyzed by tetrathionate hydrolase (TetH) yielding thiosulfate and other products [10], [24]. In addition, other RISC oxidation enzymes identified in *Acidithiobacillus* include: sulfide quinone reductase (SQR) being responsible for oxidation of hydrogen sulfide [25], and rhodanese or thiosulfate sulfurtransferase (TST) transferring a sulfur atom from thiosulfate to sulfur acceptors like cyanide and thiol compounds [26], [27]. Recently, a model of sulfur oxidation in *A. ferrooxidans* was proposed, in which electrons from oxidation of RISCs are transferred via the quinol pool (QH_2_) to terminal oxidases to produce ATP or to NADH complex I to generate NADPH, coupling the oxidation of RISCs with the generation of energy or reducing power [23].

An unique sulfur oxidation system exists in *A. caldus*, which is quite different from that of *A. ferrooxidans* according to comparative genome analysis [28]. The sulfur oxidation system of *A. caldus* can be classified into three subsystems: the truncated Sox subsystem, non-Sox sulfur subsystem, and SOR subsystem. The truncated Sox subsystem in *A. caldus* contains two copies of essential *soxABXYZ* genes but no *soxCD* genes. The non-Sox sulfur subsystem, similar to the sulfur oxidation system in *A. ferrooxidans*, contains the sulfur oxidation enzyme genes (*tetH*, *sqr*, *rhd*, and *hdrABC*) and terminal oxidase genes. The SOR subsystem is characterized by the sulfur oxygenase reductase gene (*sor*) in *A. caldus*, which was only found in several acidophilic and thermophilic archaea but not in the two species *A. ferrooxidans* and *A. thiooxidans* [10], [29], [30]. Therefore, *A. caldus* has a complex and integrated sulfur oxidation system. The currently known sulfur oxidation pathways in *A. caldus* are mainly acquired from the genome sequence analysis, which are not clear and remain unanswered questions: (1) How is the elemental sulfur oxidized and how many elemental sulfur oxidizing pathways exist? (2) How does the truncated Sox subsystem work? (3) How do the various pathways interconnect to complete the sulfur oxidation? Although, a rough model for ISCs metabolism in *A. caldus* was proposed [31], the above questions are still awaiting clear answers.

Recently, a method for *A. caldus* mutant construction was developed in our laboratory, which is different from the previous reported gene knock-out method based on the homologous recombination in *A. caldus* [32]. In our experiments, construction of gene mutant via the transposition insertional mutagenesis of the insertion sequences (IS elements) in *A. caldus* was discovered for the first time. The first report about IS element transposition in *Acidithiobacillus* was the ISAfe1 insertional inactivation of *resB* (a putative cytochrome c-type biogenesis protein) producing the *A. ferrooxidans* mutant, which lost Fe (II) oxidation ability [33], [34]. IS elements are also widely distributed in *A. caldus* [35]. The loss of the *sor* gene resulting from the ISAtc1 transposition in *A. caldus* SM-1 was proposed according to the comparison of *A. caldus* SM-1 genome sequence and the *sor* gene cloned from SM-1 [30], [35].

In this research, *A. caldus* MTH-04 *Δsor* mutant is constructed by electroporation of a suicide plasmid. The cell growth of the mutant is measured using S^0^ and K_2_S_4_O_6_ as the substrates. Then, comparative transcriptome analysis is carried out by whole-genome microarray and quantitative RT-PCR. Finally, an integrated sulfur oxidation model in *A. caldus* is proposed.

## Results and Discussion

### Construction of *Δsor* mutant

**Figure 1:**
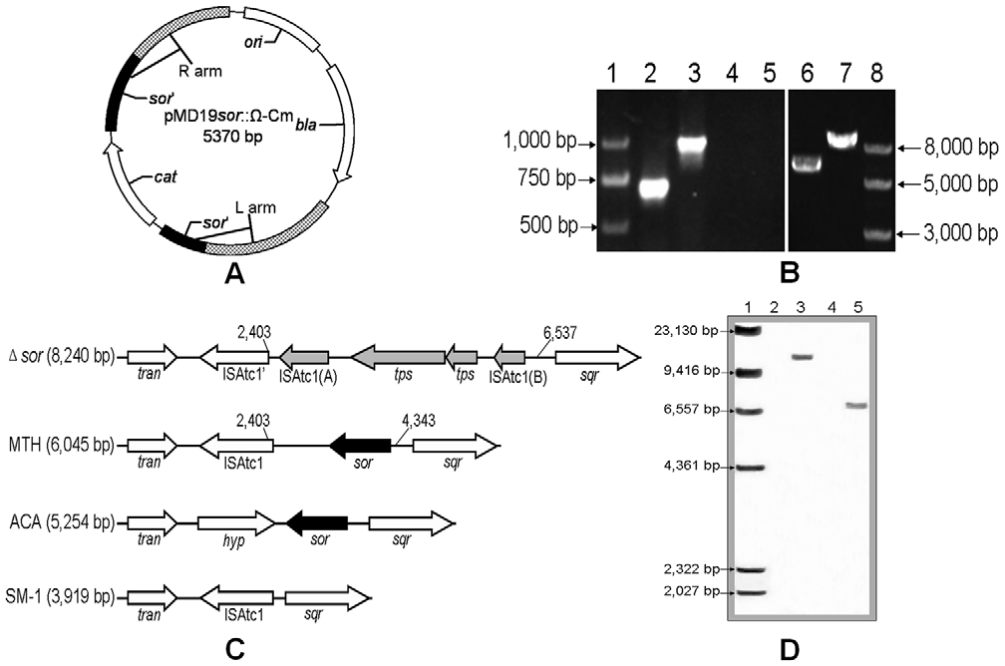
Construction of the mutant. (A) The pMD19*sor*::Ω-Cm suicide plasmid. The plasmid carries the mutant allele of *sor* gene disrupted by the chloramphenicol resistance gene (816 bp), generating two homologous sequences L-arm (1,321 bp) and R-arm (1,332 bp). (B) PCR analysis of *A. caldus* MTH-04 *sor* mutant. Lanes 4, 5 and 7, the chromosome from the mutant; lanes 2, 3 and 6, the chromosome from the wild type; lanes 2 and 4, primers of SorA fwd and SorA rev; lanes 3 and 5, primers of SorB fwd and SorB rev; lanes 6 and 7, primers of Big fwd and Big rev. (C) Comparison of the regions of the *sor* gene. *Δsor*: *A .caldus* MTH-04 *Δsor* mutant; MTH: *A .caldus* MTH-04 wild type; ACA: *A .caldus* ATCC 51765; SM-1: *A .caldus* SM-1; *tran*: ABC transporter ATP-binding protein; ISAtc1: IS elements; *tps*: transposase; *sqr*: sulfide quinone reductase; *sor*: sulfur oxygenase reductase; *hyp*: hypothetical protein. (D) Southern blot analysis of the wild type and the mutant using the *sor* probe. Lane 2, negative control with ddH_2_O; lane 3, positive control with pJRD215-tac-sor (12 kbp) digested by *Eco*R I; lane 4, the mutant chromosome digested by *Eco*R I; lane 5, the wild type chromosome digested by *Eco*R I.

The suicide plasmid pMD19*sor*::Ω-Cm (Figure 1A) derived from pUC19 is unable to replicate in *A. caldus*. It carries the homologous sequence of the disrupted *sor* gene by insertion of chloramphenicol resistance gene (*cat*, 816 bp) generating two homologous sequences, the L-arm (1,321 bp) and the R-arm (1,332 bp) (Figure 1A). *A. caldus* MTH-04 was electroporated using the suicide plasmid and the mutants were screened using colony PCR, then the chromosomes of the screened mutants were extracted and analyzed by PCR (Figure 1B) and southern blot analyses (Figure 1D). As shown in Figure 1B, two sets of primers (SorA fwd and SorA rev, SorB fwd and SorB rev) specific to the *sor* gene and a set of primers (Big fwd and Big rev) specific to the homologous sequence were used to verify the mutant. Fragments of 684 bp (lane 2, Figure 1B) and 971 bp (lane 3, Figure 1B) specific to the *sor* gene were amplified from the chromosome of the wild type, but not from the mutant. An 8 kbp fragment amplified from the mutant using the primers Big fwd and Big rev (lane 7, Figure 1B) was larger than the fragment of about 6 kbp amplified from the wild type (lane 6, Figure 1B). The fragments of lane 6 and lane 7 in Figure 1B were sequenced for validation. The results are shown in Figure 1C: the *sor* gene region (from 2,403 bp to 4,343 bp) in *A. caldus* MTH-04 wild type was different from the corresponding region (from 2,403 bp to 6,537 bp) which is replaced by the IS element (ISAtc1) and the transposase gene in the *A. caldus* MTH-04 mutant. In order to further confirm the mutant, southern blot analysis was carried out using the *sor* gene as the probe. As shown in Figure 1D, an *EcoR* I-fragment (6.7 kbp) was isolated from the wild type chromosome, but not from the mutant. In addition, the analysis of the *sor* regions in different *A. caldus* strains is depicted in Figure 1C, ISAtc1 is positioned upstream of the *sor* gene in the wild type *A. caldus* MTH-04 genome, similar to *A. caldus* SM-1 but different from *A. caldus* ATCC 51765, while the *sor* gene region in the *Δsor* mutant was replaced by the IS elements.

The hypothesis that IS element transposition results in the loss of *sor* gene in *A. caldus* SM-1 has been proposed according to sequence analysis [35]. Fortunately, it was confirmed for the first time in our experiments. Firstly, the same result was obtained in the repeated experiments and no mutant was obtained when plasmid pMD19 or pSDU1 was used, which showed that deletion of *sor* gene was not a natural occurrence. Secondly, no artificial or exogenous IS elements was introduced into the suicide plasmid. Finally, the IS element (ISAtc1) causing the loss of *sor* gene could be found on *A. caldus* MTH-04 wild type chromosome. For above reasons, it is clear that the transposition of IS elements in *A. caldus* MTH-04 chromosome leads to the loss of *sor* gene. Moreover, it also implies the role of IS elements in gene evolution and metabolic diversity in *A. caldus*.

### Growth curve analysis of the wild type and the *Δsor* mutant

**Figure 2:**
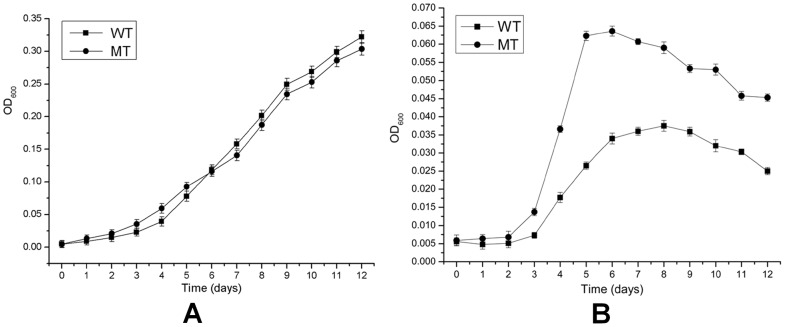
Growth curves of *A. caldus* MTH-04 and the *Δsor* mutant (A) in Starkey-S^0^ medium and (B) in Starkey-K_2_S_4_O_6_ medium. Each data point represents triplicate results. The error bars indicate standard deviations.

The growth curves of the wild type and the mutant in Starkey-S^0^ or Starkey-K_2_S_4_O_6_ liquid media are shown in Figure 2. When S^0^ was utilized as the sole substrate, the cell concentration of the mutant was a little higher than the wild type in the first six days, but was slightly lower for the next six days (Figure 2A). When K_2_S_4_O_6_ was used as the sole substrate, the mutant had an obvious growth advantage compared to the wild and its maximum cell concentration was 70% higher than the wild (Figure 2B). The mutant still had S^0^ oxidation ability, indicating that SOR is not the sole and determinative elemental sulfur oxidation pathway and there are other additional elemental sulfur oxidation pathways in *A. caldus*. In addition, the maximum value of OD_600_ of *A. caldus* in S^0^ medium in Figure 2A could reach about 0.35 much higher than that in K_2_S_4_O_6_ medium (OD_600_≈0.065), so it is suggested that *A. caldus* has a high efficient elemental sulfur oxidizing ability to enable it grow well.

### Comparative transcriptome analysis

**Figure 3:**
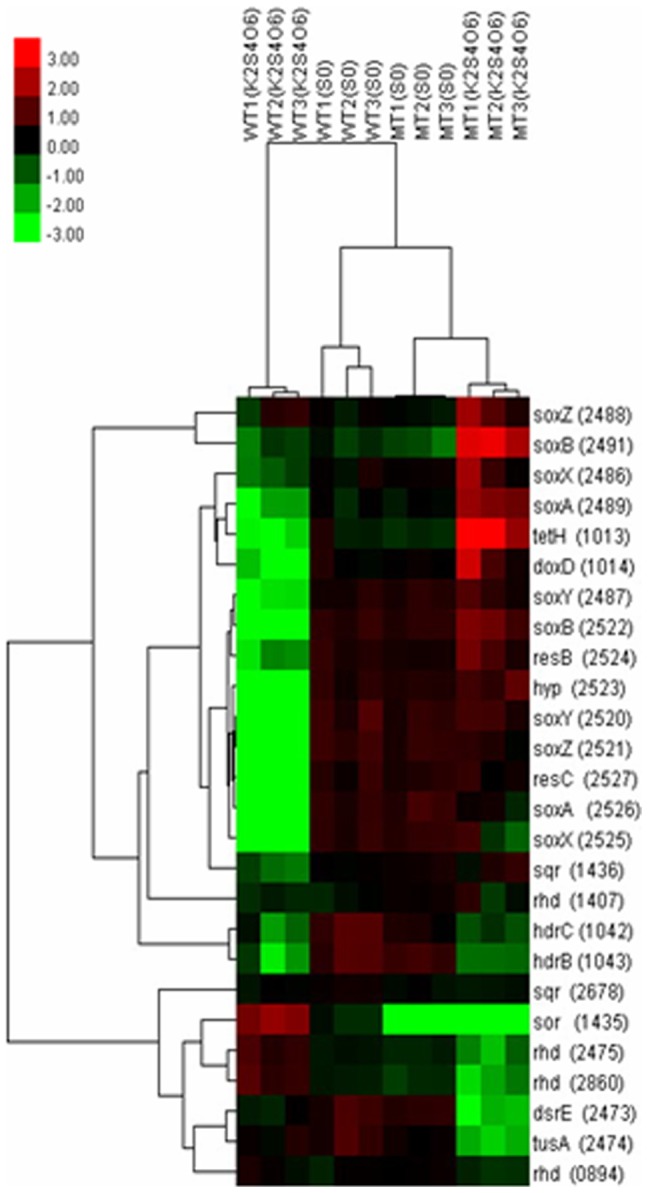
Hierarchical cluster analysis of genes involved in sulfur oxidation. The signals are shown in a red-green color scale, where red represents higher expression and green represents lower expression. Each column stands for a sample and each row stands for a gene.

Hybridization scheme, microarray experiments, and data analysis are described in materials and methods. Unsupervised hierarchical cluster analysis of sulfur oxidation genes are depicted in Figure 3. It shows the normalized transcription levels of sulfur oxidation genes for each sample compared to the common reference. Several remarkable differences are revealed in this figure: firstly, there was a bright green region related to the genes in the sox operons of the wild but a red color region of the mutant in K_2_S_4_O_6_ medium, suggesting that genes in the sox operons had a low expression level in the wild and a high expression level in the mutant when K_2_S_4_O_6_ was applied; secondly, the bright green color appeared in the row of *sor* gene of the mutant was resulted from the deletion of *sor*, moreover the *sor* gene of the wild displayed red color in K_2_S_4_O_6_ medium and green color in S^0^ medium, indicating the higher expression level of *sor* in K_2_S_4_O_6_ medium; thirdly, the green region at the lower right corner of the figure indicated that genes involving *rhd*, *dsrE*, and *tusA* had lower expression levels in the mutant when K_2_S_4_O_6_ was applied.

The results of significance analysis of microarrays (SAM) for sulfur oxidation genes under four cases are shown in Table 1 (the numbers without brackets). The expression patterns observed with microarrays were validated by using real-time quantitative RT-PCR (qRT-PCR) for some sulfur oxidation genes (Table 1, the numbers in the brackets). The results from microarrays and qRT-PCR were highly consistent. Genes that met the criterions of fold change ≥1.5 with q-value≤0.05 and fold change ≤0.667 with q-value≤0.05 were up-regulated and down-regulated, respectively. The expression of sulfur oxidation genes showed distinct differences under the four experimental cases (Table 1). In the first case, the comparison was between the mutant and the wild using S^0^ as sulfur source shown in the column of MT/WT (S^0^). No obvious difference was found in the expression levels of sulfur oxidation genes except that the expression of *sor* was not detected in the mutant. The results implied that deletion of *sor* had no obvious influence on the other sulfur oxidation pathways in S^0^ medium at the early phase. In the second case, K_2_S_4_O_6_ was used as sulfur source to analyze the differential gene expressions between the mutant and the wild. The loss of *sor* led to significant gene expression changes in K_2_S_4_O_6_ medium, in particular: (1) the remarkable up-regulation of genes in the two sox operons; (2) the significant up-regulation of genes in tetrathionate hydrolase operon (*tetH* and *doxD*); (3) the obvious down-regulation of sulfur transferase genes, such as *dsrE* (ACAL_2473), *tusA* (ACAL_2474), and *rhd* (ACAL_2475, ACAL_2860). The results showed that deletion of *sor* stimulated the up-regulation of the sox pathway and the S_4_I pathway in K_2_S_4_O_6_ medium. In the third case, the effect of different sulfur sources on the gene expression of the wild type was showed in the column of S^0^/K_2_S_4_O_6_ (WT). Sulfur oxidation genes of the two sox operons, tetrathionate hydrolase operon, and heterodisulfide reductase complex operon had higher expression levels of varied degrees when S^0^ was added, in contrast, *sor* had a lower expression level. Therefore, the lower expression level of *sor* gene at the early fast growth phase in S^0^ medium and the better growth of the mutant at the early days in S^0^ medium implied that SOR does not play an important role at the early stage of the elemental sulfur oxidation process. The fourth case showed the differential gene expressions of the mutant in the two kinds of media. An obvious change compared to the third case was that the majority of sulfur oxidation genes of sox operons and tetrathionate hydrolase operon had higher expression levels in K_2_S_4_O_6_ medium than that in S^0^ medium. From above results together with the following facts that the lower expression levels of these genes of the wild type in the K_2_S_4_O_6_ medium (Table 1, column of S^0^/K_2_S_4_O_6_ (WT)) and the no differential expression of these genes between the mutant and the wild in S^0^ medium (Table 1, column of MT/WT (S^0^)), it can be concluded that there was a significant increase of the expression levels of above genes in the mutant compared with that in the wild type in K_2_S_4_O_6_ medium. Above conclusion showed that deletion of SOR pathway stimulated the sox and S_4_I pathways in K_2_S_4_O_6_ medium.

**Table 1:** Expression of sulfur oxidation genes under different cases.

ID	Gene	Function	MT/WT (S^0^)	MT/WT (K_2_S_4_O_6_)	S^0^/K_2_S_4_O_6_ (WT)	S^0^/K_2_S_4_O_6_ (MT)
Sox operon I						
ACAL_2486	*soxX*	cytochrome c class I	NC (1.41±0.02)	3.86±1.03 (23.88±0.05)	2.19±0.03 (17.21±0.01)	NC (0.18±0.04)
ACAL_2487	*soxY*	sulfur covalently binding protein	NC (0.97±0.06)	12.92±0.08 (14.27±0.04)	10.89±0.01 (14.40±0.05)	NC (0.17±0.03)
ACAL_2488	*soxZ*	Sulfur compound chelating protein	NC (1.40±0.03)	1.96±0.86 (27.43±0.04)	NC (14.68±0.02)	0.43±0.76 (0.13±0.01)
ACAL_2489	*soxA*	cytochrome c (diheme)	NC (1.43±0.03)	13.68±0.14 (6.02±0.04)	4.35±0.02 (3.32±0.01)	0.33 ±0.14 (0.14±0.04)
ACAL_2491	*soxB*	sulfate thiol esterase	NC (1.39±0.03)	10.64±1.30 (11.15±0.06)	NC (4.90±0.04)	0.09±1.30 (0.11±0.01)
Sox operon II and cytochrome *bo_3_* ubiquinol oxidase						
ACAL_2515	*coxB*	cytochrome c oxidase, subunit II	NC	5.40±0.01	18.36±0.02	3.65±0.02
ACAL_2516	*cox*	cytochrome c oxidase	NC	22.26±0.01	53.67±0.05	2.36±0.03
ACAL_2517	*cyoC*	cytochrome quinol oxidase subunit 3	NC	9.61±0.01	25.37±0.02	2.32±0.01
ACAL_2518	*hyp*	hypothetical protein	NC	11.24±0.01	33.64±0.01	2.34±0.01
ACAL_2519	*hyp*	hypothetical protein	NC	13.93±0.05	10.40±0.01	NC
ACAL_2520	*soxY*	sulfur covalently binding protein	NC (1.48±0.01)	29.24±0.04 (20.59±0.02)	29.98±0.06 (174.46±0.03)	NC (2.18±0.01)
ACAL_2521	*soxZ*	sulfur compound chelating protein	NC (1.32±0.01)	17.12±0.03 (35.92±0.02)	21.94±0.01 (31.38±0.06)	NC (0.20±0.01)
ACAL_2522	*soxB*	sulfate thiol esterase	NC (1.66±0.03)	34.24±0.17 (74.87±0.01)	21.80±0.01 (180.30±0.04)	0.61±0.18 (0.70±0.02)
ACAL_2523	*hyp*	hypothetical protein	NC	31.41±0.07	24.64±0.02	NC
ACAL_2524	*resB*	cytochrome c-type maturation protein	NC	7.04±0.21	5.36±0.02	NC
ACAL_2525	*soxX*	cytochrome c class I	NC (1.42±0.04)	10.06±0.21 (15.11±0.05)	17.66±0.01 (222.40±0.02)	1.81±0.21 (3.62±0.01)
ACAL_2526	*soxA*	cytochrome c (diheme)	NC (1.60±0.02)	22.44±0.02 (117.45±0.05)	32.03±0.01 (182.13±0.06)	1.55±0.05 (0.43±0.04)
ACAL_2527	*resC*	cytochrome c-type maturation protein	NC	17.33±0.04	19.03±0.03	NC
Tetrathionate hydrolase operon						
ACAL_1013	*tetH*	tetrathionate hydrolase	NC (1.00±0.03)	57.02±7.02 (83.40±0.02)	7.73±0.05 (38.06±0.01)	0.10±0.13 (0.08±0.03)
ACAL_1014	*doxD*	Thiosuirate quinine oxidoreductase subunit	NC (1.15±0.06)	18.41±2.74 (14.12±0.02)	8.91±0.02 (50.22±0.01)	0.46±0.10 (0.71±0.03)
Sulfur oxygenase reductase						
ACAL_1435	*sor*	sulfur oxygenase reductase	0.00±0.00 (0.00±0.01)	0.00±0.08 (0.00±0.04)	0.26±0.08 (0.51±0.02)	UD (UD)
Sulfide-quinone reductase						
ACAL_1436	*sqr*	sulfide quinone reductase	NC (2.64±0.04)	2.66±0.06 (1.01±0.05)	2.24±0.01 (3.06±0.01)	NC (1.40±0.01)
ACAL_2678	*sqr*	sulfide quinone reductase	0.83±0.01	NC	NC	NC
Heterodisulfide reductase complex operon						
ACAL_1042	*hdr*C	heterodisulfide reductase subunit C	0.65±0.06 (0.94±0.02)	NC (0.42±0.03)	3.77±0.10 (27.60±0.06)	2.01±0.02 (10.76±0.01)
ACAL_1043	*hdr*B	heterodisulfide reductase subunit B	NC (1.08±0.02)	NC (0.59±0.04)	5.74±0.09 (41.14±0.01)	3.78±0.01 (1.55±0.03)
ACAL_2473	*dsrE*	hypothetical protein (sulfur transferase)	NC (0.91±0.04)	0.20±0.01 (0.21±0.03)	1.76±0.06 (9.93±0.02)	7.97±0.00 (7.51±0.01)
ACAL_2474	*tusA*	hypothetical protein (sulfur transferase)	0.72±0.01 (1.12±0.02)	0.20±0.02 (0.14±0.01)	NC (3.86±0.04)	4.86±0.01 (5.43±0.05)
ACAL_2475	*rhd*	rhodanese (sulfur transferase)	NC (0.89±0.01)	0.21±0.04 (0.20±0.03)	0.56±0.04 (0.85±0.05)	2.40±0.01 (2.02±0.01)
Rhodanese (sulfur transferase)						
ACAL_0894	*rhd*	rhodanese (sulfur transferase)	NC	NC	NC	NC
ACAL_1407	*rhd*	Rhodanese (sulfur transferase)	1.27±0.01	NC	NC	NC
ACAL_2860	*rhd*	rhodanese (sulfur transferase)	NC (1.04±0.04)	0.16±0.05 (0.15±0.01)	0.50±0.05 (1.66±0.02)	2.72±0.01 (1.99±0.03)
Cytochrome *bd* ubiquinol oxidase						
ACAL_0179	*cydA*	cytochrome d ubiquinol oxidase, subunit I	NC	0.59±0.05	NC	NC
ACAL_0180	*cydB*	cytochrome d ubiquinol oxidase, subunit II	NC	2.09±0.15	2.32±0.03	NC
ACAL_1110	*cydB*	cytochrome d ubiquinol oxidase, subunit II	0.61±0.01	1.66±0.01	3.66±0.01	NC
ACAL_1111	*cydA*	cytochrome d ubiquinol oxidase, subunit I	NC	6.25±0.01	6.44±0.02	NC
ACAL_1252	*cydB*	cytochrome d ubiquinol oxidase, subunit II	NC	2.42±0.14	2.59±0.03	NC
ACAL_1253	*cydA*	cytochrome d ubiquinol oxidase, subunit I	NC	1.86±0.15	NC	NC
ACAL_2185	*cydA*	cytochrome d ubiquinol oxidase, subunit I	UD	UD	UD	UD
ACAL_2186	*cydB*	cytochrome d ubiquinol oxidase, subunit II	UD	UD	UD	UD
ACAL_2017	*cydB*	cytochrome d ubiquinol oxidase, subunit II	NC	2.09±0.04	2.02±0.02	NC
Cytochrome *bo_3_* ubiquinol oxidase						
ACAL_1757	*cyoB*	cytochrome o ubiquinol oxidase, subunit II	NC	3.31±0.01	9.61±0.01	2.79±0.01
ACAL_1758	*hyp*	hypothetical protein	NC	NC	3.33±0.11	3.69±0.02
ACAL_1759	*cyoA*	cytochrome o ubiquinol oxidase, subunit I	NC	15.28±0.01	30.69±0.00	1.76±0.01
ACAL_1760	*cyoC*	cytochrome o ubiquinol oxidase, subunit III	0.73±0.08	2.16±0.01	9.27±0.00	3.11±0.01
ACAL_1761	*hyp*	hypothetical protein	0.74±0.05	5.96±0.00	21.87±0.00	2.71±0.02
ACAL_1762	*cyoD*	cytochrome o ubiquinol oxidase, subunit IV	NC	10.49±0.00	25.81±0.00	2.40±0.01
Cytochrome c protein						
ACAL_0446	*resC*	cytochrome c-type maturation protein	NC	20.67±0.21	5.34±0.00	0.24±0.21
ACAL_1072	*resC*	cytochrome c-type maturation protein	NC	17.35±0.21	2.54±0.01	0.13±0.09
NADH complex I operon						
ACAL_0727	*nuoA*	NADH ubiquinone oxidoreductase A subunit	NC	NC	NC	NC
ACAL_0728	*nuoB*	NADH ubiquinone oxidoreductase B subunit	NC	0.61±0.08	0.67±0.01	NC
ACAL_0729	*nuoC*	NADH ubiquinone oxidoreductase C subunit	NC	0.43±0.06	0.35±0.03	NC
ACAL_0730	*nuoD*	NADH ubiquinone oxidoreductase D subunit	NC	0.60±0.04	NC	1.79±0.03
ACAL_0731	*nuoE*	NADH ubiquinone oxidoreductase E subunit	NC	0.71±0.00	NC	1.57±0.01
ACAL_0732	*nuoF*	NADH ubiquinone oxidoreductase F subunit	NC	2.15±0.02	2.88±0.01	NC
ACAL_0733	*nuoG*	NADH ubiquinone oxidoreductase G subunit	NC	3.66±0.03	3.68±0.11	NC
ACAL_0734	*nuoH*	NADH ubiquinone oxidoreductase H subunit	NC	3.35±0.11	3.15±0.10	NC
ACAL_0735	*nuoI*	NADH ubiquinone oxidoreductase I subunit	NC	NC	0.38±0.54	ND
ACAL_0736	*nuoJ*	NADH ubiquinone oxidoreductase J subunit	NC	NC	NC	NC
ACAL_0737	*nuoK*	NADH ubiquinone oxidoreductase K subunit	NC	NC	1.88±0.04	1.35±0.02
ACAL_0738	*nuoL*	NADH ubiquinone oxidoreductase L subunit	ND	ND	ND	ND
ACAL_0739	*nuoM*	NADH ubiquinone oxidoreductase M subunit	ND	ND	ND	ND
ACAL_0740	*nuoN*	NADH ubiquinone oxidoreductase N subunit	NC	6.29±0.15	5.53±0.02	NC
ATP synthetase complex operon						
ACAL_2147	*atpB*	ATP synthase F0, A subunit	NC	0.34±0.60	0.26±0.55	NC
ACAL_2148	*atpE*	ATP synthase F0, C subunit	NC	NC	NC	NC
ACAL_2149	*atpF*	ATP synthase F0, B subunit	NC	NC	NC	1.53±0.01
ACAL_2150	*atpH*	ATP synthase F1, delta subunit	NC	0.52±0.48	NC	1.67±0.01
ACAL_2151	*atpA*	ATP synthase F1, alpha subunit	0.74±0.01	NC	2.49±0.23	1.37±0.01
ACAL_2152	*atpG*	ATP synthase F1, gamma subunit	0.79±0.01	NC	1.54±0.19	NC
ACAL_2153	*atpD*	ATP synthase F1, beta subunit	NC	NC	2.59±0.15	1.94±0.05
ACAL_2154	*atpC*	ATP synthase F1, epsilon subunit	NC	4.94±0.03	4.56±0.12	NC

Fold Change ≥1.5, q-value≤0.05: up-regulation, Fold Change ≤0.667, q-value≤0.05: down-regulation;

NC: not credible, q-value>0.05;

UD: undetected;

Numbers without brackets from microarrays; numbers in brackets from qRT-PCR.

### Model of the sulfur oxidation system in *A. caldus*

#### The role of SOR in elemental sulfur oxidation

**Figure 4:**
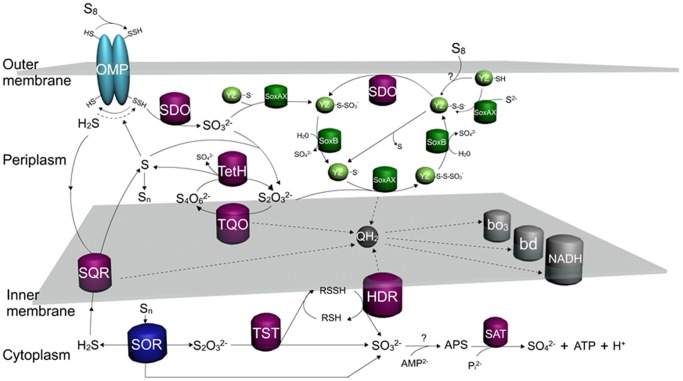
Model of sulfur oxidation in *A. caldus*. The sulfur oxidation system involves varied sulfur oxidation pathways and the electron transfer system in different cellular compartments. Starting from the extracellular elemental sulfur (S_8_), it is activated and transported into the periplasmic space as persulfide sulfur (R-SH), and then oxidized by the sulfur dioxygenase (SDO) to produce SO_3_^2−^; SO_3_^2−^ can enter into Sox pathway or combine with sulfur atoms to form S_2_O_3_^2−^ via a nonenzymatic reaction; S_2_O_3_^2−^ has two destinies, one is to be oxidized by the Sox pathway, the other is to form S_4_O_6_^2−^ catalyzed by thiosuirate quinine oxidoreductase (TQO); S_4_O_6_^2−^ is hydrolyzed by tetrathionate hydrolase (TetH) producing S_2_O_3_^2−^, SO_4_^2−^, and S; S produced from hydrolysis of S_4_O_6_^2−^, oxidation of H_2_S by sulfide quinone reductase (SQR) or from truncated oxidation of S_2_O_3_^2−^ by the Sox pathway can be accumulated in the form of polymeric sulfur (S_n_) in the periplasm and transferred into the cytoplasm; the cytoplasmic elemental sulfur (S_n_) is oxidized by sulfur oxygenase reductase (SOR) producing S_2_O_3_^2−^, SO_3_^2−^, and H_2_S, which stimulate the cytoplasmic sulfur pathways including the metabolism of S_2_O_3_^2−^ by rhodanese (TST) and heterodisulfidereductase (HDR) and the oxidation of SO_3_^2−^ via the APS pathway. Two methods of SoxYZ regeneration are proposed, with one being the sulfur atom is provided from the sulfane intermediate (SoxYZ–S–S^−^) and the other being oxidation of SoxYZ–S–S^−^ by SDO to complete the Sox sulfur oxidation pathway. Electrons from SQR, TQO, HDR and SoxAX are mediated by the quinol pool in the inner membrane, then are utilized by terminal oxidases *bd* or *bo_3_* to produce a proton gradient to generate ATP or by the NADH complex I to generate reducing power.

Our experimental data can be explained when SOR is considered a cytoplasmic enzyme oxidizing elemental sulfur in the cytoplasm without coupling with the electron transfer chain or substrate-level phosphorylation in *A. caldus*. A hypothetical model is shown in Figure 4, sulfur atoms (S) produced from other sulfur oxidation pathways in the periplasm can be accumulated in form of polymeric sulfur (S_n_), then S or S_n_ are transported via an unknown mechanism into the cytoplasm where they are immediately oxidized by SOR. The higher expression level of *sor* gene in K_2_S_4_O_6_ medium can be explained by cytoplasmic located SOR. K_2_S_4_O_6_ is much easily and quickly entering into the periplasm, where it is hydrolyzed by TetH to produce sulfur atoms and transported into the cytoplasm inducing the expression of *sor*. While S^0^ needs to be activated before being transported into the periplasm, which is slower than that of K_2_S_4_O_6_, resulting in the lower expression level of *sor* in S^0^ medium. The role of SOR is supported by the facts that the *sor* (ACAL_1435) gene that encodes a sulfur oxygenase reductase with the ability to oxidize S^0^, has been cloned and expressed in *E. coli* and the enzymatic activity of S^0^ oxidation has been detected without addition of glutathione in our laboratory (unpublished data). The location of SOR in cytoplasm is in agreement with the reports that there are no intracellular sulfur globules in *A. caldus*, whereas sulfur globules are accumulated in *A. ferrooxidans* which acks *sor* gene [36], and SOR is reported existed in the cytoplasm in archaea [10].

SOR is not coupled with the electron transfer chain or substrate-level phosphorylation. The obvious growth advantage of the *Δsor* mutant in the first six days in S^0^ medium or during the whole growth period in K_2_S_4_O_6_ medium (Figure 2) should be caused by the sulfur oxidation pathway shifting from SOR to other pathways coupled with the electron transfer chain. Moreover, it is not an efficient way to produce electrons for sulfur atoms to be oxidized by SOR when sulfur atoms in cytoplasm are insufficient caused by the delay of sulfur activation and transportation at the early growth stage in S^0^ medium. In contrast, when sulfur atoms are sufficient at the late growth stage in S^0^ medium, SOR oxidizes the sulfur atoms to produce other sulfur compounds, which enter other sulfur oxidation pathways in the cytoplasm to produce electrons. These could be the reason for the lowered cell growth of the *Δsor* mutant in S^0^ medium than the wild type after six days of cultivation (Figure 2A).

SOR plays a central role in the cytoplasmic sulfur oxidation pathways. The possible products from the SOR catalyzed reactions are thiosulfate, sulfite and sulfide [17], [37]. Thiosulfate is considered to be catalyzed by rhodanese (TST) in the cytoplasm of *A. caldus* according to the reasons: (1) TST widely exists in the cytoplasm of both prokaryotes and eukaryotes, and it transfers a sulfur atom from thiosulfate to thiol compounds producing persulfide and sulfite [27], [38]; (2) there is an obvious relevance between the expressions of *sor* and rhodanese (*rhd*) genes. As shown in Figure 3, when K_2_S_4_O_6_ was applied, the expressions of *sor* and *rhd* (ACAL_2475, ACAL_2860) showed red and dark colors, respectively, for the wild type, while the expressions of the two *rhd* genes showed bright green for the *Δsor* mutant; when S^0^ was applied, the expressions of the two *rhd* genes showed a little brighter for the *Δsor* mutant than that for the wild. Above results showed that the expressions of the two *rhd* genes down-regulated when *sor* was deleted. Consequently, a hypothetical thiosulfate oxidation pathway was put forward and shown in Figure 4: the thiol proteins (RSH) in the cytoplasm of *A. caldus* can be used as sulfur atom acceptors for the catalysis of thiosulfate by TST, producing sulfane sulfate (RSSH) which is used as the substrate of the heterodisulfide reductase complex (HDR), thus a cycle is formed, in which RSH obtains a sulfur atom to form RSSH catalyzed by TST and then RSSH is oxidized by HDR to regenerated RSH. Sulfite is toxic to the cell and needs to be oxidized rapidly. An APS (adenosine-5′-phosphosulfate) pathway for sulfite oxidation maybe exists in *A. caldus* which is similar to the sulfite oxidation pathway in *A. ferrooxidans* [23]. A putative sulfate adenylyltransferase dissimilatory-type/denylylsulfate kinase (*sat*) gene was discovered in *A. caldus* MTH-04 but the APS reductase gene that catalyzed sulfite to adenosine-5′-phosphosulfate (APS) was not determined (see Figure 4). Sulfide could be converted to hydrogen sulfide and then oxidized by sulfide:quinine reductase (SQR) located in the cytoplasmic membrane. Therefore, it is presumed that SOR plays a central role in the cytoplasmic elemental sulfur oxidation in *A. caldus*.

#### Other elemental sulfur oxidation pathways

An important elemental sulfur oxidation pathway in the periplasm near the outer membrane of *A. caldus* is hypothesized as shown in Figure 4: the extracellular elemental sulfur (S_8_) is activated by thiol groups of special outer-membrane proteins and transported into the periplasmic space as persulfide sulfur (R-SH), and then oxidized by the sulfur diooxygenase (SDO), meanwhile hydrogen sulfide is produced during the activation of S_8_. Sulfur diooxygenase gene (*sdo*) in the *A. caldus* MTH-04 genome was discovered, cloned and expressed in *E. coli* in our laboratory, and the enzymatic activity of SDO was detected with the addition of glutathione (data to be published). Although several outer-membrane proteins related to sulfur oxidation in *A. ferrooxidans* have been reported, the thiol-bearing membrane proteins are not yet identified [39], [40]. Another proposed elemental sulfur oxidation pathway locating in the cytoplasm near the inner membrane is shown in Figure 4: heterodisulfide reductase (HDR) catalyzes sulfane sulfate (RSSH) to produce sulfite and regenerate RSH. Two subunits genes (*hdrB* and *hdrC*) of HDR were found in the draft genome sequence of *A. caldus* MTH-04 but all genes (*hdr*A, *hdrB* and *hdrC*) of HDR were found in *A. caldus* ATCC 51756 [31].

In summary,there are three elemental sulfur oxidation pathways in *A. caldus*: (1) the pathway based on SDO in the periplasm; (2) the pathway based on the HDR in cytoplasm near the inner membrane; and (3) the pathway based on the SOR in the cytoplasm.

#### The Sox pathway

The truncated Sox pathway in *A. caldus* MTH-04 contains two *sox* gene clusters, *sox* operon I (*soxX-soxY-soxZ-soxA-hyp-soxB*) and *sox* operon II (*coxB-cox-cyoC-hyp-hyp-soxY-soxZ-soxB-hyp-resB-soxX-soxA-resC*). There is also a strong connection between the expressions of the *sox* genes and the terminal oxidase genes. As shown in the columns MT/WT (K_2_S_4_O_6_) and S^0^/K_2_S_4_O_6_ (WT) in Table 1, the expression of terminal oxidase genes, especially the cytochrome *bo_3_* ubiquinol oxidase genes, upregulated significantly when *sox* genes had high expression levels. The well-studied Sox pathway in *P. pantotrophus* demonstrated that it couples the sulfur oxidation with the electron transfer [8]. Besides, the *sox* operon II together with a *bo_3_* ubiquinol oxidase operon make up a big gene cluster on the chromosome, which implies the feasible regulation and control on their transcriptional level. The hypothetical pathway is shown in Figure 4: electrons produced from the Sox system via QH_2_ were transferred to the terminal oxidases (*bd* and *bo_3_*) and the NADH complex to produce ATP and NADPH, respectively. However, an important question is raised as to how the truncated Sox system works in *A. caldus* in the absence of SoxCD. Two possible ways to regenerate SoxYZ are put forward. One way is that the sulfur atom of the sulfane intermediate (SoxYZ–S–S^−^) is dropped from SoxYZ, which has been reported in other bacteria [10], [41], [42]. Another way is that the sulfur atom of the sulfane intermediate is oxidized by sulfur dioxygenase (SDO), because SoxY is actually a kind of thiol-bearing protein that may be used as an oxidation substrate of SDO. In addition, SoxY is presumed to play a role in the activation of S_8_ for the reasons that elemental sulfur (S_8_) assembled on the thiol of SoxY via nonenzymatic conjugation has been reported [10], [42], and the expression of *soxY* gene of the wild type is much higher in S^0^ medium than that in K_2_S_4_O_6_ medium (Table 1). Therefore, the Sox system in *A. caldus* has capabilities to oxidize a wide range of sulfur compounds including sulfite, thiosulfate, sulfide and elemental sulfur and couple the process with electron transfer, which make the Sox pathway play a central role in the periplasmic sulfur oxidation system.

#### The S_4_I pathway

The reaction cycle linking the hydrolysis of tetrathionate and the oxidation of thiosulfate is an important sulfur oxidation pathway in the periplasm of *A. caldus*. Tetrathionate hydrolase (TetH), a soluble periplasmic enzyme, is responsible for the hydrolysis of tetrathionate in *A. caldus* [43]. One of the hydrolysates of tetrathionate catalyzed by TetH is thiosulfate, but the other products are uncertain [43], [44]. The higher expression level of the *sor* gene in K_2_S_4_O_6_ medium than that in S^0^ medium (Table 1) implied that sulfur atoms (S) may be one of the products [44]. Thiosulfate quinine oxidoreduetase (TQO), which is constituted of subunits DoxA and DoxD, catalyzes thiosulfate producing tetrathionate. The expression levels of *tetH* and *doxD* of the wild type in S^0^ medium were much higher than that in K_2_S_4_O_6_ medium (Table 1), suggesting that a mass of S_4_O_6_^2−^ and S_2_O_3_^2−^ were produced during elemental sulfur oxidation. Therefore, the pathways are proposed: S_2_O_3_^2−^ is synthesized by nonenzymatic reaction from sulfite and a sulfur atom, then oxidized by TQO, producing S_4_O_6_^2−^ in the cytomembrane (Figure 4). However, only *doxD* gene was found in all published *A. caldu*s genomes, while *doxA* was not found. The thiosulfate oxidation process by TQO was illustrated in *A. ambivalens*: DoxD catalyzes S_2_O_3_^2−^ to S_4_O_6_^2−^ and gains two electrons, meanwhile, DoxA transfers electrons to the quinine [45]. Both subunits are thought to be constituents of the terminal oxidase and the function of DoxA is to transfer electrons, so it is hypothesized that other terminal oxidase in place of DoxA combines with DoxD to constitute TQO in *A. caldus*. In addition, due to the instability of S_2_O_3_^2−^ and the stability of S_4_O_6_^2−^ in the acidic environment, the produced S_2_O_3_^2−^ in the periplasms is rapidly oxidized by the Sox system or entered into the S_4_I pathway. For this reason, formation of S_4_O_6_^2−^ via the S_4_I pathway may be important for sulfur storage.

#### Electron transfer from sulfur oxidation to respiratory system

Little is known about the electron transfer chain in *A. caldus*, but it has been well studied in *A. ferrooxidans*. One of the electron transfer chains in *A. ferrooxidans* is that electrons from Fe (II) oxidation flow through Cyc2 to rusticyanin, and then reduce oxygen via a cytochrome *aa_3_* complex (downhill pathway) or reduce NAD^+^ via a *bc*1/quinone pool/NADH complex (uphill pathway) [23], [46], [47]. The other one is that electrons from RISCs oxidation are transferred via the quinol pool (QH_2_) either to terminal oxidases *bd*, *bo_3_* and *aa_3_* to produce a proton gradient, or to NADH complex I to generate reducing power [23], [46]. *A. caldus* only has the sulfur oxidation system and its genome information shows that four copies of *bd* ubiquinol oxidase genes (*cydAB*), two gene clusters encoding *bo_3_* ubiquinol oxidase (*coxB* and *cyoC*) and a cytochrome c oxidase gene (*cox*, ACAL_2516) exist in the *A. caldus* MTH-04 draft genome. SoxAX, a c-type cytochrome complex that consists of SoxA as a diheme subunit and SoxX as a monoheme [48], can carry two electrons during the catalyzing process in the Sox pathway. The comparative transcriptome analysis showed that once the expression of genes in Sox pathway upregulated, the expression of *bd* and *bo_3_* oxidases genes were also upregulated (Table 1), which implied that electrons could be transported from the Sox pathway to *bd* and *bo_3_* oxidases in *A. caldus*. Therefore, a hypothesized electron transfer chain based on the quinol pool in *A. caldus* is depicted in Figure 4: electrons from SQR, TQO, HDR and SoxAX are transferred via the quinol pool either directly to terminal oxidases *bd* or *bo_3_* to produce the proton gradient to generate ATP, or directly to NADH complex I to generate reducing power. Furthermore, there are several features of the electron transfer in *A. caldus* including: (1) SoxAX connects the Sox pathway with the electron transfer chain, so that makes the Sox pathway become an important sulfur oxidation pathway for producing mass electrons; (2) Quinol terminal oxidases (*bd* and *bo_3_*) are the dominant terminal oxidases as more electrons are produced from S^0^ oxidation than that from Fe (II) oxidation; (3) The quinol pool (QH_2_) located in the cytoplasmic membrane may play a regulatory role in the electron transfer chain. Overall, a powerful electron transfer and respiratory system including the quinol pool (QH_2_), terminal oxidases and NADH complex exist in *A. caldus*.

### Conclusions

The growth measurements of the wild type and the mutant, and the comparative transcriptome analysis extend our understanding of the complicated sulfur oxidation system in *A. caldus*. Several distinctive features of the sulfur oxidation system of *A. caldus* are summarized as follows: (1) The sulfur oxidation system of *A. caldus* involves the Sox subsystem, a non-Sox sulfur subsystem similar to that in *A. ferrooxidans*, and SOR which work together to complete the oxidation of elemental sulfur and various sulfur compounds, so that *A. caldus* has a growth advantage over any other bacteria in the bio-mining reactor [35]. (2) Regional division of the sulfur oxidation system in *A. caldus* is an obvious characteristic. The first region is the outer membrane of the periplasm where the extracellular elemental sulfur (S_8_) is activated and transported into the periplasmic space as persulfide sulfur(R-SH). The second region is the periplasm where the Sox pathway, SDO and TetH perform their functions, and this is an important place for sulfur oxidation. Moreover, nonenzymatic reactions producing thiosulfate from sulfite and sulfur atoms and forming polysulfur (S_n_) are complementary to the periplasmic sulfur oxidation system. The third region is the cytoplasmic membrane involving SQR, TQO and HDR with a common feature of coupling sulfur oxidation with electron transport. The fourth region is the cytoplasm with an SOR-based sulfur oxidation system. (3) There are complex controls on the sulfur oxidation process. On one hand, the sulfur oxidation related genes belong to different pathways regulated at the transcriptional level to adapt to the production and consumption of various sulfur compounds during the process of elemental sulfur oxidation. On the other hand, metabolic control on the substrate level may be an important regulatory method, as the poly-sulfur (S_n_) and poly-thionates (S_4_O_6_^2−^) accumulated in the periplasm could be the main forms for energy storage.

In summary, an integrated sulfur oxidation model of *A. caldus* is proposed based on comparative transcriptomic analysis, which provides new insights and guides for the future study of the sulfur oxidation metabolism. In view of the diversity of the RISCs and the complexity of the sulfur oxidation system of *A. caldus*, many fundamental questions such as identification of sulfur oxidation genes and determination of enzyme activities remain to be resolved. Fortunately, the establishment of genetic manipulation of *A. caldus* provides effective and powerful tools for elucidation of the sulfur oxidation mechanism of *A. caldus* [32], [49], [50].

## Methods

### Bacteria and culture conditions

The bacterial strains and plasmids used are listed in Table 2. The media of Luria broth (LB) or agar plate for *E. coli* were described in reference [51]. Liquid Starkey-S^0^ and Starkey-K_2_S_4_O_6_ inorganic medium and solid Starkey-Na_2_S_2_O_3_ medium for cultivation of *A. caldus* MTH-04 were prepared as described in reference [52]. Elemental sulfur (S^0^) (boiling sterilized, 20 g/L) and K_2_S_4_O_6_ (membrane filtration, 5 g/L) was added before inoculation. Chloromycetin (Cm) was added to the final concentrations of 34 µg/ml in LB medium, 60 µg/ml in liquid Starkey-S0 medium and solid Starkey-Na_2_S_2_O_3_ medium, respectively. The cultivation temperatures were 37°C for *E. coli* and 40°C for *A. caldus* MTH-04. The shaking speed for liquid cultivation of *A. caldus* MTH-04 was 125 r/min if not specifically stated.

**Table 2:** Bacterial strains and plasmids used in this study.

Strain or plasmid	Genotype or description	Source or reference
*A. caldus* MTH-04	Isolated from Tengchong area, Yunnan province, China	[58]
*A. caldus* MTH-04 *Δsor* mutant	*Δsor*	This study
*E. coli DH5α*	F^−^φ80d *lacZΔ*M15*Δ*(*lacZYA-argF*) U169 *end A1 recA1 hsdR17*(r_k_^−^,m_k_^+^) *supE44λ-thi-1 gyr96 relA1 phoA*	TransGen Biotech Corp. China
Plasmids		
pSIMPLE19 EcoR V/BAP	Ap^r^; lacZ′; ColE1 replicon; blunt-tailed PCR product cloning vector	TaKaRa Biotechnology Co. China
pSIMPLE19*hsdM*::Ω-Cm	Ap^r^ Cm^r^; suicide plasmid containing the *hsdM* gene inserted by the chloromycetin resistance gene	Our laboratory
pSDU1	Cm^r^; IncQ; mob^+^	Our laboratory
pJRD215-tac-sor	Sm^r^, Km^r^; IncQ, Mob^+^; tac promoter;*sor*	Our laboratory
pMD19*sor*::Ω-Cm	Ap^r^ Cm^r^; suicide plasmid containing the *sor* gene inserted by the chloromycetin resistance gene	This study

### Generation of mutant

The suicide plasmid was constructed as follows. The essential part of the suicide plasmid was amplified from pSIMPLE19*hsdM*::Ω-Cm using the primers of PMD fwd and PMD rev, then digested with *Sal* I and *Not* I. A 2.6 kbp homologous sequence arm holding the *sor* gene was amplified from *A. caldus* MTH-04 chromosome using the primers of Whol fwd and Whol rev, digested with *Sal* I and *Not* I, and ligated to the essential part of the suicide plasmid. The resulting plasmid was named pMD19*sor*, then linearized in the middle of the *sor* gene, using the primers of Mid fwd and Mid rev. The linear plasmid pMD19*sor* was digested with *Kpn* I and *Bgl* II, and ligated to the chloramphenicol resistance gene (*cat*) amplified from plasmid pSDU1 using primers of Cat fwd and Cat rev digested with *Kpn* I and *Bgl* II. The generated plasmid carrying the mutant allele of *sor* disrupted by the *cat* was the suicide plasmid pMD19*sor*::Ω-Cm. The suicide plasmid was sequenced by Invitrogen Corp. for sequence confirmation. The sequences of the primers are listed in Table 3. The restriction enzymes and Prime STAR HS Taq were purchased from TaKaRa Corp. The suicide plasmid was electroporated into *A. caldus* MTH-04 using the methods described in the reference [49].

**Table 3:** Primers used in constructing the suicide plasmid and the mutant.

Name	Sequence
PMD fwd	ATAAGAATGCGGCCGCGCGGTAATACGGTTATCCAC
PMD rev	TCCGGAATTCGCGTCGACAATGGTTTCTTAGACGTCAGGT
Wholfwd	TCCGGAATTCGCGTCGACGTGAACACCGTGATCTTGTCC
Whol rev	ATAAGAATGCGGCCGCTCACAGTTCCGACGTTTTCAGT
Mid fwd	TCGGGGTACCGTTCCGATTTACCCAGGATCA
Mid rev	TTGGAAGATCTCTTCGATGACCATATCGAGACAG
Cat fwd	CTGAAGATCTTCATGTTTGACAGCT
Cat rev	TCGGGGTACCATTCATCCGCTTATTATCACTT
Clnfwd	TCCGCTCGAGGGTACCGTCTTAGAGCAAAGCGCCTGT
Cln rev	CTAGTCTAGAGCCGTAATCGGCGGAGTTTAT
SorAfwd	ATCCCATGGCCGGTCGTTAT
SorA rev	TGGTTGTGGATTTGTCGCAG
SorBfwd	ATCAGCTTGGAGGCAATGTG
SorB rev	ATGGTCTTGGGCTCTTGGTC
Big fwd	CGAGTCCCGCCCATTGTT
Big rev	TACAGGTAGAAGGCTTCACC

Colony PCR was used to screen the mutants using the primers of Clnfwd and Cln rev. The mutants were incubated in liquid Starkey-S^0^ medium, and then collected to extract the chromosome. The chromosomes of the mutants were analyzed using PCR. Firstly, two sets of primers (SorA fwd and SorA rev, SorB fwd and SorB rev) specific to the *sor* gene were used to verify the mutants. Then, PCR amplification was done using primers of Big fwd and Big rev specific to the mutant allele using *LA taq* (TaKaRa Corp.) and the amplified fragments were sequenced by Invitrogen Corp. for sequence confirmation. The sequences of the primers used are listed in Table 3.

Southern hybridization was done to further confirm the mutants. Genomic DNA of the wild type and the mutants were extracted using a QIAmp® DNA Mini kit (Qiagen Corp.), digested with *EcoR*I(NEB Corp.), electrophoresed on agarose gel, and transferred by capillary blotting to positively charged Hybond-N membranes (Roche Corp.). The *sor* probe was obtained by incorporation of alkali-labile DIG-dUTP (Roche Corp.) during PCR elongation with primers SorAfwd and SorA rev. Hybridization was carried out under stringent conditions as recommended by the manufacturer.

### Growth measurements of the wild type and the *Δsor* mutant

*A. caldus* MTH-04 and the *Δsor* mutant were inoculated into 150 ml of fresh Starkey-S^0^ medium with bacterial loading at 10% (vol/vol). Cells were harvested at the stationary growth phase. The solid sulfur in the cultivation broth was removed by low-speed centrifugation (100× g) before cell harvesting. Then, the cells were harvested by high-speed centrifugation (10,000× g), washed twice using deionized distilled H_2_O (ddH_2_O), and diluted to the final concentration with OD_600_ at 1.0. An aliquot (400 µl) of the diluted cells was inoculated into 150 ml Starkey-S^0^ or Starkey-K_2_S_4_O_6_ medium and incubated at 40°C, 125 r/min. Each condition was replicated three times and the medium without inoculation was used as the control. An aliquot (250 µl) of each sample was taken, stand for 5 minutes, and then 200 µl was taken from each of the 250 µl sample and used for OD measurement using a Microplate Spectrophotometer (Molecular Devices) at the wavelength of 600 nm. Samples were taken and measured every 24 hours until the 12^th^ day.

### Construction of the whole-genome microarray

An *A. caldus* genome 60 bp oligonucleotide microarray was obtained from CapitalBio Corporation (Beijing, China) [53]. Briefly, the *A. caldus* genome oligonucleotide set consisting of 5′ amino acid-modified 60 bp probes and representing 3,603 ORFs was synthesized by Biosune Corporation. The 3,603 ORFs consist of 2,972 ORFs from *A. caldus* MTH-04 and 631 ORFs from *A. caldus* ATCC 51756 (GenBank: ACVD00000000). Then oligonucleotides were dissolved in EasyArray™ spotting solution (CapitalBio Corp.) at 40 µM concentration, and printed in triplicate on PolymerSlide, in which the surface is covered by a thin layer of aldehyde group modified three-dimensional polymer chain (CapitalBio Corp.). On the slide, there are 48 blocks and each block has 18 columns and 15 rows.

### Design of the hybridization scheme on the gene chips

A complex hybridization scheme was designed using two-color platforms and common reference samples to determine the levels of the differential expressions between the group pairs [53]. As shown in Table 4, there were four experimental groups: WT (S^0^): the wild type in S^0^ medium, WT (K_2_S_4_O_6_): the wild type in K_2_S_4_O_6_ medium, MT (S^0^): the mutant inS^0^ medium, and MT (K_2_S_4_O_6_): the mutant in K_2_S_4_O_6_ medium. Each group contained triplicate biological repeats, so there were totally 12 samples. An aliquot of each sample was taken, mixed and used as common reference. Samples labeled with Cy5-dCTP and common reference labeled with Cy3-dCTP were hybridized on a chip. The gene expression level in the samples was determined based on the common reference. Therefore, there were four cases based on group pairs, which were the gene differential expression in S^0^ medium between the mutant and the wild type (MT/WT (S^0^)), the gene differential expression in K_2_S_4_O_6_ medium between the mutant and the wild type (MT/WT (K_2_S_4_O_6_)), the gene differential expression of the wild type between S^0^ medium and K_2_S_4_O_6_ medium (S^0^/K_2_S_4_O_6_ (WT)), and the gene differential expression of the mutant between S^0^ medium and K_2_S_4_O_6_ medium (S^0^/K_2_S_4_O_6_ (MT)).

**Table 4:** The hybridization scheme on the gene chips.

Number	Hybridization on the chip	Group	Ratio
1	WT1(S^0^) vs reference		
2	WT2(S^0^) vs reference	WT(S^0^)	
3	WT3(S^0^) vs reference		
4	WT1(K_2_S_4_O_6_) vs reference		
5	WT2(K_2_S_4_O_6_) vs reference	WT (K_2_S_4_O_6_)	(1) MT/WT (S^0^)
6	WT3(K_2_S_4_O_6_) vs reference		(2) MT/WT (K_2_S_4_O_6_)
7	MT1(S^0^) vs reference		(3) S^0^/K_2_S_4_O_6_ (WT)
8	MT2(S^0^) vs reference	MT (S^0^)	(4) S^0^/K_2_S_4_O_6_(MT)
9	MT3(S^0^) vs reference		
10	MT1(K_2_S_4_O_6_) vs reference		
11	MT2(K_2_S_4_O_6_) vs reference	MT (K_2_S_4_O_6_)	
12	MT3(K_2_S_4_O_6_) vs reference		

Note: W(S^0^): the wild type in Starkey-S^0^ medium with three repeats W1(S^0^), W2(S^0^) and W3(S^0^); W(S_4_): the wild type in Starkey-K_2_S_4_O_6_ medium with three repeats W1(S_4_), W2(S_4_) and W3(S_4_); M(S^0^): the *Δsor* mutant in Starkey-S^0^ medium with three repeats M1(S^0^), M2(S^0^) and M3(S^0^); M(S_4_): the *Δsor* mutant in Starkey-K_2_S_4_O_6_ medium with three repeats M1(S_4_), M2(S_4_) and M3(S_4_); reference:the mixed RNA of the 12 samples.

### RNA isolation and microarray experiments

The wild type and the *Δsor* mutant were cultured as described in the section of growth measurements of the wild type and the *Δsor* mutant. Cells were collected at exponential growth phase (4^th^ day), washed twice using sterile RNase-free ddH_2_O and treated with RNA protect Bacteria Reagent (Qiagen Corp.). Total RNA was extracted using RNeasy Mini Kit (Qiagen Corp.) in accordance with the manufacturer's instructions.

Total RNA was purified using Nucleospin® RNA Clean-up (MN Corp.) and tailed with polyA using Poly (A) Polymerase (Ambion Corp.). The tailed RNA was used to produce cy5/cy3-labeled cDNA employing an RNA amplification strategy (CapitalBio eukaryotic RNA Amplification and Labeling Kit, CapitalBio Corp.). Cy5/cy3-labeled cDNA were hybridized with the microarray at 42°C for 16 h. Following hybridization, the arrays were washed using two consecutive solutions (0.2% SDS, 2× SSC at 42°C for 5 min, and 0.2× SSC for 5 min at room temperature). Arrays were scanned using a confocal LuxScan™scanner and the images obtained were then analyzed using LuxScan™ 3.0 software (both from CapitalBio Corp.). For individual channel data extraction, faint spots with intensities below 400 units after background subtraction in both channels (Cy3 and Cy5) were removed. A space- and intensity-dependent normalization based on a LOWESS program was employed [54]. The raw data were Log 2 transformed and median centered by arrays and genes using the Adjust Data function of CLUSTER 3.0 software and then further analyzed using hierarchical clustering with average linkage [55]. To determine the significant differentially expressed genes whose ratio changes ≥1.5 fold with p≤0.05, Significance Analysis of Microarrays (SAM, version 3.02) was performed using two-class unpaired comparison in the SAM procedure [56].

### Real-time quantitative RT-PCR (qRT-PCR)

Total RNA was extracted as described above and was treated with RNase-Free DNase Set (Qiagen Corp.) to eliminate the traces of genomic DNA. The total RNA of 2 µg was reversely transcribed using M-MLV Reverse Transcriptase (Invitrogen Corp.) under the following conditions: 25°C for 10 min, 37°C for 60 min, and 70°C for 10 min. RT reaction products of 1 µl were used for PCR amplification using Power SYBR Green PCR Master Mix (Applied Biosystems). The conditions for the PCR reaction were as follows: 95°C for 10 min followed by 40 cycles at 95°C for 15 s and 60°C for 1 min using a 7900 HT Fast RealTime PCR system (Applied Biosystems). The glyceraldehyde-3-phosphate dehydrogenase gene (*gapdh*) was used as the reference gene for normalization. The relative expression was calculated using the comparative ΔΔC_T_ method, and the values were expressed as 2^−ΔΔCT^ [57]. Primers used in this study are listed in Table 5.

**Table 5:** Primers used in qRT-PCR.

Primers		Sequence
soxX(2486)	Fwd	GGTCGGGCTATTGCCTTTG
	Rev	TGGTCTGGAACATCTGCTGG
soxY(2487)	Fwd	CGTATCGCCCAAGGTGAAG
	Rev	CCAGACGGTGCTGACGTAATC
soxZ(2488)	Fwd	GTGGAAGTCCGCTCCCTGA
	Rev	ACGCTGACGGTCTGAATGAAG
soxA(2489)	Fwd	CAGACCCTCAAAGAATCCCG
	Rev	CTCCACCTGATGAGTCTTGTTGTC
soxB(2491)	Fwd	AGGACCCATACACCATACCTGAC
	Rev	TTGAGGACGGAGCCTACGC
soxY(2520)	Fwd	ACAGCATTGGCAAGACCTCC
	Rev	AACTCACCCTTGTTCGTCCG
soxZ(2521)	Fwd	CAAACCCCTGTGCCATCTG
	Rev	ATTGTCCTTCCAAGTCATCGC
soxB(2522)	Fwd	GGATTACTACGGCATCAAAGCA
	Rev	ACCCAACTGTTCACGATAGCG
soxX(2525)	Fwd	GTACAGGCGGGCAATGTTG
	Rev	CGTCAAGACCTTATCCTTACCAAA
soxA(2526)	Fwd	CTATCAGCAGTATCAAGAGGCAATC
	Rev	CGGAAAGCAGGACGCATAG
tetH(1013)	Fwd	GGCTTCAACGCCAAAACTG
	Rev	GGCATCGTAGTCCGAGGTCA
doxD(1014)	Fwd	TGTATGCTGGCGTCATTATCTTTA
	Rev	CGAAACCATCCCTTCTCCG
sor(1435)	Fwd	ACGGTGTATCGCCCTTGGT
	Rev	GGATTCGCTCCTAAAGTCGC
sqr(1436)	Fwd	TTTTATCCAGGCGGAAGTGC
	Rev	TTGTTCCCATAGTAGAAATCGGTG
hdrC(1042)	Fwd	GTACCCTGACGGACTACGAGC
	Rev	GCCCAAGCGGATGAGGTAG
hdrB(1043)	Fwd	AGCTCGTGCTCAATATCCTCC
	Rev	TGATGCCAAATTCCACTTCG
drsE(2473)	Fwd	AAGCAAAATGAAGGCGAAGG
	Rev	CGAAGAGGTCCACCGTCATC
tusA(2474)	Fwd	TCCTGAAAGTCGTTGCCACC
	Rev	TTCCGCCTGATCCAGAAGC
rhd(2475)	Fwd	CGGGCAGAATTTTCACCTCA
	Rev	CCGCTCAGACAATAGAACACGA
rhd(2860)	Fwd	GGCTCATCAACGACCACGA
	Rev	CGGCAGATACTTACCCACCTC
gapdh(2603)	Fwd	ACGTCTCCATCGTCGATCTCA
	Rev	AGGGCTTGTCGTTGTAGGCA
